# Supplementary material for: Reconciling Mining with the Conservation of Cave Biodiversity: A Quantitative Baseline to Help Establish Conservation Priorities
Source: PLoS One. 2016 Dec 20;11(12):e0168348. doi: 10.1371/journal.pone.0168348 (PMC5173368; doi:10.1371/journal.pone.0168348)
Supplement: S1 Dataset — (ZIP) [file pone.0168348.s002.zip › Taxa/Serra Norte/SN_2013/N4WS/Lista N4WS-14.pdf]

CAVIDADE N4WS-0014

Classe	Ordem	Fam/Outros	Gên/Outros	Espécie	S-U	U-S	Zon.
Annelida	Oligochaeta			jovem	2	2	E
Arachnida	Acari	(Parasitengonina)		sp.4		X	E
Arachnida	Acari	Neothyridae	*Diplothyrus*	sp.1		X	E
Arachnida	Araneae	Araneidae		jovem	X		E
Arachnida	Araneae	Corinnidae		jovem	1		E
Arachnida	Araneae	Dipluridae		jovem	1		E
Arachnida	Araneae	Drymusidae	*Drymusa*	jovem	X		E
Arachnida	Araneae	Filistatidae		sp.1	X		E
Arachnida	Araneae	Mysmenidae		jovem	X		E
Arachnida	Araneae	Ochyroceratidae	*Speocera*	sp.	X	X	E
Arachnida	Araneae	Pholcidae	*Leptopholcus*	sp.1		X	E
Arachnida	Araneae	Pholcidae	*Mesabolivar*	sp.	X		E
Arachnida	Araneae	Pholcidae	*Metagonia*	sp.	X		E
Arachnida	Araneae	Prodidomidae	*Lygromma*	sp.	X		E
Arachnida	Araneae	Salticidae		sp.	X		E
Arachnida	Araneae	Theraphosidae		sp.	X		E
Arachnida	Araneae	Theridiidae		sp.	X		E
Arachnida	Araneae	Theridiosomatidae	*Plato*	sp.1	X		E
Arachnida	Opiliones	Sclerosomatidae	*Gagrellinae*	sp.	X		E
Arachnida	Pseudoscorpiones	Chernetidae	*Spelaeochernes*	sp.1	X	X	E
Arachnida	Pseudoscorpiones	Chthoniidae	*Pseudochthonius*	sp.1		X	E
Diplopoda	Spirostreptida	Pseudonannolenidae	*Pseudonannolene*	jovem		1	E
Entognatha	Collembola	Sminthuroidea		sp.2		X	E
Entognatha	Diplura	Campodeidae		sp.1		X	E
Insecta	Blattodea			jovem	X	3	E
Insecta	Coleoptera	Carabidae		sp.13	X	X	E
Insecta	Coleoptera	Staphylinidae	Pselaphinae	sp.	X		E
Insecta	Diptera	Cecidomyiidae	Cecidomyiinae	sp.		X	E
Insecta	Diptera	Drosophilidae	*Drosophila*	*eleonorae*		X	E
Insecta	Diptera	Psychodidae	Phlebotominae	sp.		X	E
Insecta	Diptera	Sciaridae		jovem		X	E
Insecta	Diptera			sp.	X		E
Insecta	Heteroptera	Veliidae		sp.	X	X	E
Insecta	Homoptera	Cixiidae		jovem	X		E
Insecta	Hymenoptera	Formicidae	*Camponotus*	*crassus*	X		E
Insecta	Hymenoptera	Formicidae	*Pachycondyla*	*constricta*	X		E
Insecta	Hymenoptera	Formicidae	*Paratrechina*	sp.2		X	E
Insecta	Lepidoptera	Tineoidea		jovem	X		E
Insecta	Orthoptera	Phalangopsidae	*Phalangopsis*	sp.		1	E
Insecta	Psocoptera	Psocomorpha		jovem	X		E
Insecta	Thysanura	Nicoletiidae	Nicoletiinae	sp.1			E
Gastropoda	Pulmonata	Systrophiidae	*Happia*	sp.	X		E
Malacostraca	Isopoda	Armadillidae		sp.1	X		E
Malacostraca	Isopoda	Philosciidae		sp.1	X	X	E
Malacostraca	Isopoda	Scleropactidae	*Circoniscus*	*buckupi*	X		E
Symphyla		Scutigerellidae	*Hanseniella*	sp.	X		E
Amphibia	Anura			sp.		2	E
Mammalia	Chiroptera	Emballonuridae	*Peropteryx*	sp.		1	E
